# The Progression of Cardiac Damage in the Offspring of Mothers with Gestational Diabetes Is Regulated by the p53/miR-34/SIRT1/7 Pathway

**DOI:** 10.3390/ijms27104368

**Published:** 2026-05-14

**Authors:** Guadalupe Díaz-Rosas, Omar Gómez-Acuña, Renata Saucedo, Ricardo Chávez-García, Alfonso Reyes-López, Alejandra Contreras-Ramos, Clara Ortega-Camarillo

**Affiliations:** 1 Laboratorio de Investigación en Biología Molecular, Hospital Infantil de México Federico Gómez (HIMFG), Mexico City 06720, Mexico; gudiro2@yahoo.com.mx (G.D.-R.);; 2 Unidad de Investigación Médica en Enfermedades Endocrinas, Hospital de Especialidades, Centro Médico Nacional Siglo XXI, Instituto Mexicano del Seguro Social, Mexico City 06720, Mexico; renata.saucedo@imss.gob.mx; 3 Department of Agricultural and Animal Production, Universidad Autonoma Metropolitana, Xochimilco, Mexico City 04960, Mexico; 4 Centro de Estudios Económicos y Sociales en Salud del Hospital Infantil de México Federico Gómez (HIMFG), Mexico City 06720, Mexico; 5 Unidad de Investigación Médica en Bioquímica, Hospital de Especialidades, Centro Médico Nacional Siglo XXI, Instituto Mexicano del Seguro Social, Mexico City 06720, Mexico

**Keywords:** fetal programming, cardiac remodeling, cardiomyopathy, hyperglycemia, cardiomyocyte

## Abstract

Gestational diabetes mellitus (GDM) exposes the fetus to chronic hyperglycemia, promoting early cardiac remodeling and increasing the risk of diabetic cardiomyopathy later in life. Epigenetic regulators such as *p53* tumor suppressor gene (*p53*), *microRNA-34a* (*miR-34a)*, and the sirtuins 1 and 7 (SIRT1/SIRT7) may contribute to this programming process; however, their temporal dynamics during postnatal cardiac development remain unclear. This study aimed to characterize structural and molecular alterations in the hearts of offspring exposed to GDM and to determine the involvement of the *p53*–*miR-34a*–SIRT1/SIRT7 axis in early cardiac remodeling. Cardiac morphometry was assessed at birth (newborn [NB]) and at 8, 15, 25, and 35 days. Left ventricles were examined through hematoxylin/eosin staining. SIRT1, SIRT7, Bcl-2, and Bax were evaluated by immunofluorescence, while *p53* and *miR-34a* were evaluated by RT-PCR. Molecular interactions were integrated using IPA software, version 159584291. Offspring exposed to GDM exhibited a reduced cardiac area and ventricular lumen, along with increased left ventricular wall thickness and fibrosis during early postnatal stages. The cardiomyocyte area was elevated at all ages. The level of *miR-34a* increased early, preceding p53 upregulation. SIRT1 presences decreased from NB to 35 days, whereas SIRT7 expression remained consistently elevated. These findings suggest that GDM induces early and sustained cardiac remodeling associated with dysregulation of the *p53–miR-34a*–SIRT1/SIRT7 axis, a pattern that could increase susceptibility to diabetic cardiomyopathy.

## 1. Introduction

When fetal development occurs in an environment of maternal hyperglycemia, as in the case of gestational diabetes mellitus (GDM), multiple alterations are observed in both the fetus and the mother, with long-term and short-term repercussions [1].

Gestational diabetes mellitus (GDM) allows us to investigate the molecular mechanisms through which the intrauterine environment alters fetal development, consistent with the Developmental Origins of Health and Disease (DoHaD) framework [2,3]. Fetal exposure to hyperglycemia, fetal hyperinsulinemia, inflammation, and oxidative stress in GDM leads to mitochondrial dysfunction, impaired nutrient transport, and altered endocrine signaling, creating a microenvironment that directly disrupts cardiac organogenesis [4,5]. As a result, children exposed to GDM are more likely to develop ventricular hypertrophy, diastolic dysfunction and contractility abnormalities, and they are at a higher risk of cardiovascular disease in later life [3,5]. It has been reported that fetal hyperinsulinemia stimulates cardiomyocyte proliferation and glycogen deposition in the intraventricular septum (IVS) and ventricular walls, causing myocardial hypertrophy [6]. Echocardiography in fetuses exposed to GDM demonstrated a significant increase in the thickness of the posterior wall of the left ventricle (LV) and the IVS [7]. Furthermore, fetal metabolic overload and oxidative stress induce apoptosis in cardiac cells, altering fetal myocardial relaxation and contributing to diastolic dysfunction [7]. Of the molecular mediators involved in this adverse cardiac programming, *p53* tumor suppressor gene (*p53*), *microRNA-34a* (*miR-34a*), and the sirtuins 1 and 7 (SIRT1/SIRT7) have emerged as pivotal regulatory nodes. The p53 protein plays a key role in regulating cellular damage responses, modulating apoptosis and cell cycle arrest. It also plays essential roles in embryonic development and normal cardiac function [8]. However, under pathological cardiovascular conditions, p53 overexpression has been associated with antiangiogenic effects, apoptosis, and cell cycle arrest. Oxidative stress induced by maternal hyperglycemia has been shown to activate p53 and cardiomyocyte apoptosis [9]. It contributes to the development of cardiovascular diseases, especially postinfarction cardiac remodeling, as well as hypertrophic, dilated, and diabetic cardiomyopathy [10]. These findings demonstrate that p53 plays a key role as a modulator of the transition from compensatory hypertrophy to pathological hypertrophy, promoting myocardial fibrosis processes and contributing to the development of heart failure [11]. p53 activation depends on posttranslational modifications such as phosphorylation and/or acetylation [12], as well as by microRNAs [13]. Although the removal of acetyl groups from p53 mainly occurs through the action of sirtuin 1 (SIRT1), other NAD+-dependent deacetylases, such as SIRT7, have also been proposed as potential p53 regulators [14].

This family (SIRT1 to SIRT7) regulates important metabolic pathways related to glucose homeostasis, cell survival, senescence, proliferation, apoptosis, DNA repair, and caloric restriction [15]. In the heart, SIRT1 protects cardiomyocytes from oxidative stress damage during ischemia–reperfusion, but sustained activation can promote cardiac hypertrophy [16]. Another sirtuin involved in cardiac homeostasis is SIRT7; its inhibition activates p53, triggers cardiomyocyte apoptosis, and leads to hypertrophy [17]. Sirtuin activity is modulated by microRNAs. A regulatory loop among *p53*, SIRT1, and *miR-34a* [18,19] has been described, in which *p53* induces *miR-34a* and *miR-34a* suppresses SIRT1, amplifying pro-apoptotic signaling—especially under oxidative or genotoxic stress, partly through Bax overexpression [19]. However, SIRT7 and p53 interaction is context-dependent, meaning that the effects of this interaction can be either protective or pathological, depending on the tissue type and metabolic condition [14]. Despite these advances, the interaction between p53, SIRT1, SIRT7 and miR-34a during the early postnatal period and its impact on the development of cardiac damage in offspring of mothers with gestational diabetes remains unclear.

This study analyzes the timing of cardiac damage in the offspring of mothers with gestational diabetes, with an emphasis on the left ventricle. This finding suggests the existence of a positive regulatory cycle mediated by *p53*, SIRT1/7, and *miR-34*, which could influence the progression of diabetic cardiomyopathy during the fetal period. Integrated analysis of *p53*/SIRT1/7/*miR-34* will provide a deeper understanding of the epigenetic mechanisms underlying structural and functional cardiac damage, thus contributing to a better understanding of pathogenesis and the identification of potential therapeutic targets to prevent cardiovascular complications associated with fetal programming in the context of maternal hyperglycemia.

## 2. Results

### 2.1. Postnatal Left Ventricular Hypertrophy Is a Consequence of Gestational Diabetes Mellitus

**Figure 1:**
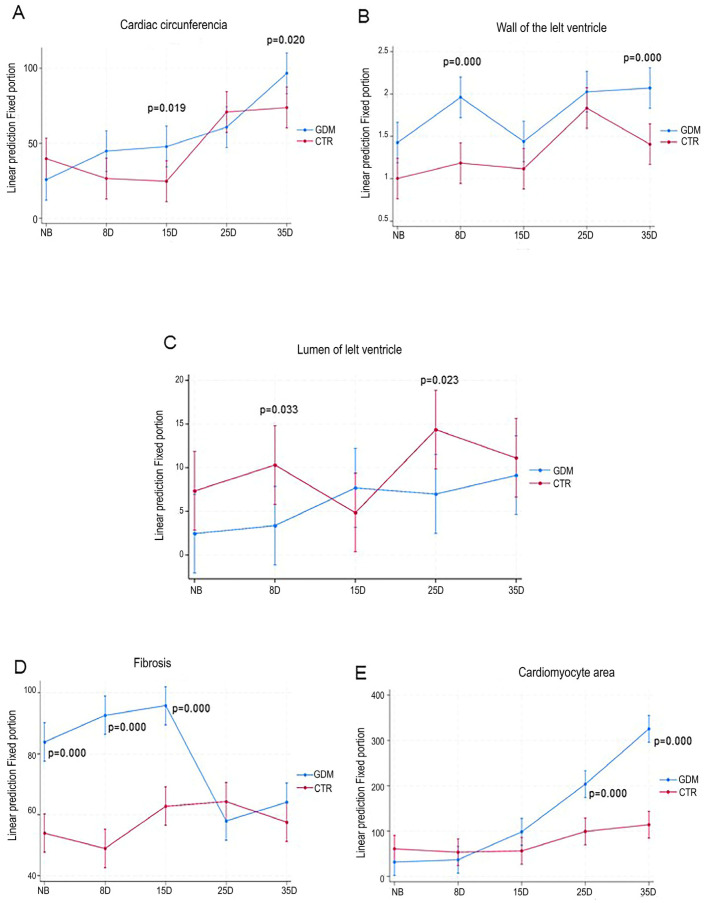
Effects of gestational diabetes on the heart of rat pups during the postnatal period. Notably, morphological analyses revealed the development of left ventricular hypertrophy (LVH) up to day 15 (15D), as evidenced by a smaller heart area (**A**). Furthermore, the left ventricular wall thickens (**B**), while the lumen narrows (**C**), accompanied by fibrosis (**D**) and cardiomyocyte growth (**E**) The data are expressed as the mean ± SD; *n* = 5 litters per group.

The newborn offspring of mothers with GDM showed a non-significant reduction in total heart area (approximately 30%) compared with the control offspring. From day 8 to day 35, heart size progressively increased in GDM offspring, with statistically significant differences observed on days 15 and 35 when compared with CTR offspring (*p* = 0.02, CTR; Figure 1A).

### 2.2. GDM Increases Left Ventricular Wall Thickness (LVWL) and Reduces the Left Ventricular Lumen in Offspring

Compared with that in the control offspring, the LVWL in the offspring of mothers with GDM increased. However, statistically significant differences were observed only in the 8D and 35D groups (Figure 1B; *p* < 0.000). On the other hand, the total area of the left ventricular lumen was significantly reduced in GDM offspring. Significant reductions were observed on days 8 and 25 compared with the control group (*p* < 0.05; Figure 1C).

### 2.3. Myocytes Are Greater in the Offspring of Mothers with GDM

The myocyte area increased significantly in the offspring of mothers with GDM from day 15 onwards. This effect was most pronounced on days 25 and 35 (*p* < 0.001). Compared with control offspring, the myocyte area on day 25 was approximately doubled, and on day 35, it had increased nearly threefold (Figure 1E). Myocyte hypertrophy showed a moderate correlation with LVWL (r = 0.521; *p* = 0.00) and strong correlation with heart circumference (r = 0.749; *p* = 0.00), indicating that cellular enlargement occurred alongside the progression of cardiac structural remodeling.

### 2.4. GDM Increases the Fibrosis Index During the First Few Days After Birth

**Figure 2:**
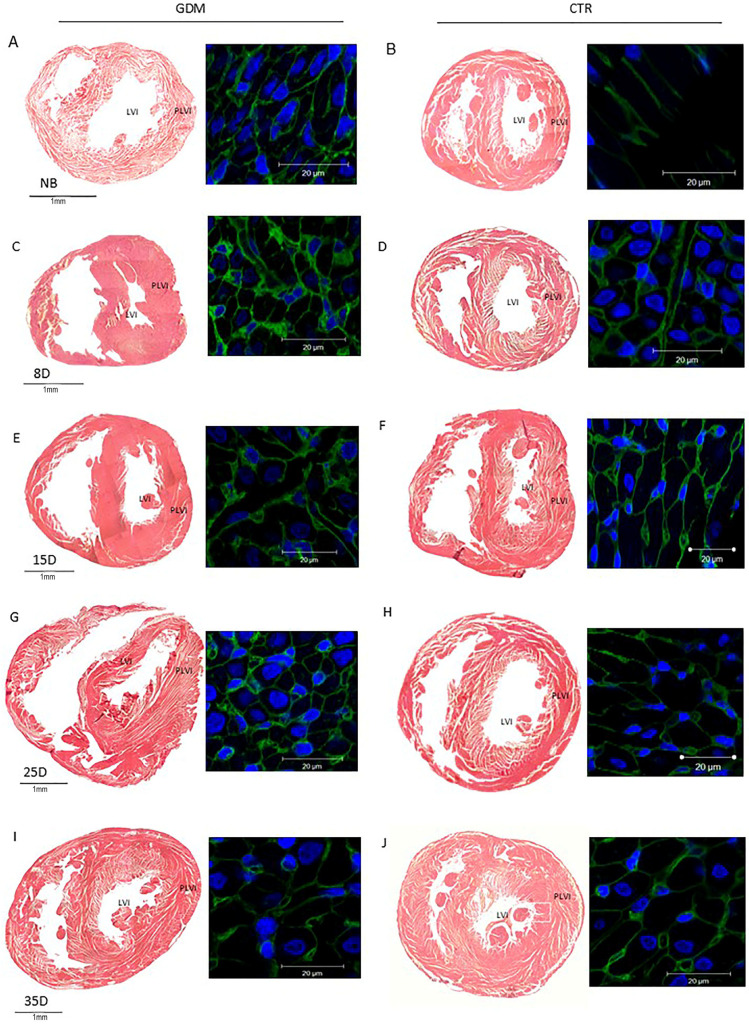
Mosaic of transverse heart micrographs from the offspring of rats with gestational diabetes (**left column**) and control rats (**right column**). The images were stained with haematoxylin and eosin, WGA (green) and DRAQ7 (blue, nuclei). Panels (**A**,**B**) show newborns (NB); panels (**C**,**D**) show 8-day-olds (8D); panels (**E**,**F**) show 15-day-olds (15D); panels (**G**,**H**) show 25-day-olds (25D); and panels (**I**,**J**) show 35-day-olds (35D). Show changes in left ventricular wall thickness and cardiomyocyte growth (WGA stain) in the GDM group compared to the control group. *n* = 5 litters per group.

Cardiac damage in the offspring with GDM was assessed based on the disorganization of cardiac fibers. The fibrosis index was significantly different among the offspring of mothers with GDM. Cardiac damage was observed primarily in newborns and persisted in 8D and 15D offspring (Figure 1E and Figure 2; *p* < 0.000) compared with the control group. The extent of myocardial fibrosis reduced in 25D and 35D GDM offspring, reaching values similar to those in the control offspring. Furthermore, the progression of fibrosis was negatively correlated with the reduction in the ventricular lumen (r = −0.374; *p* = 0.011).

### 2.5. Expression of Postnatal p53 and miR-34a in the Left Ventricle of Offspring Following Developmental GDM Exposure

**Figure 3:**
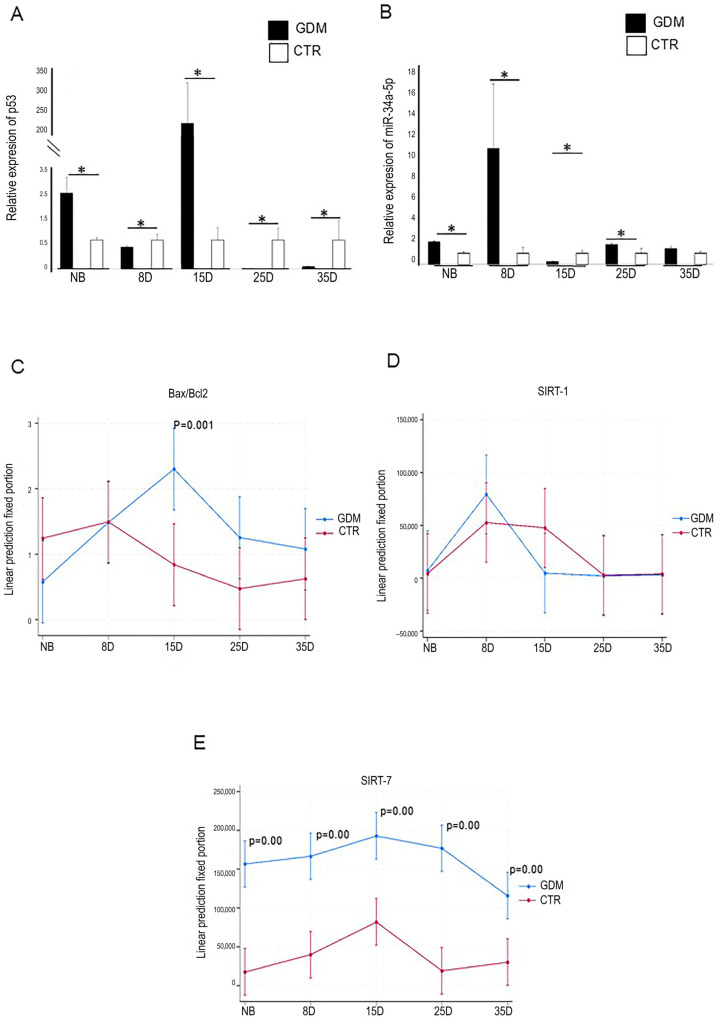
Evaluation of the p53/miR-34/SIRT1/7 axis in postnatal hearts from CTR and GDM pups. p53 mRNA increased at NB and on day 15 (**A**), miR-34a on day 8 (**B**), and the Bax/Bcl-2 ratio on day 15 (**C**). SIRT1 without changes (**D**); SIRT7 pixel intensities were significantly higher in GDM pups (**E**). Mean ± SD; *n* = 5 litters per group; * *p* < 0.05 vs. CTR.

The GDM group had significantly higher *p53* mRNA expression levels in their hearts when compared to the control group. The levels were 1.5-fold higher at birth and approximately 200-fold higher at 15 days (*p* < 0.001; Figure 3A). In contrast, *p53* expression in 8-, 25-, and 35-day-old GDM offspring was lower than in their respective control groups. In terms of *miR-34a*, there was a minor rise in expression in the offspring of GDM subjects at both 25 days and at birth. However, a significant surge was seen on day 8 in comparison to all other time points in both the CTR and GDM groups (*p* < 0.001; Figure 3B).

### 2.6. GDM Promotes Apoptosis-Regulating Proteins in the Heart Tissue of Offspring

**Figure 4:**
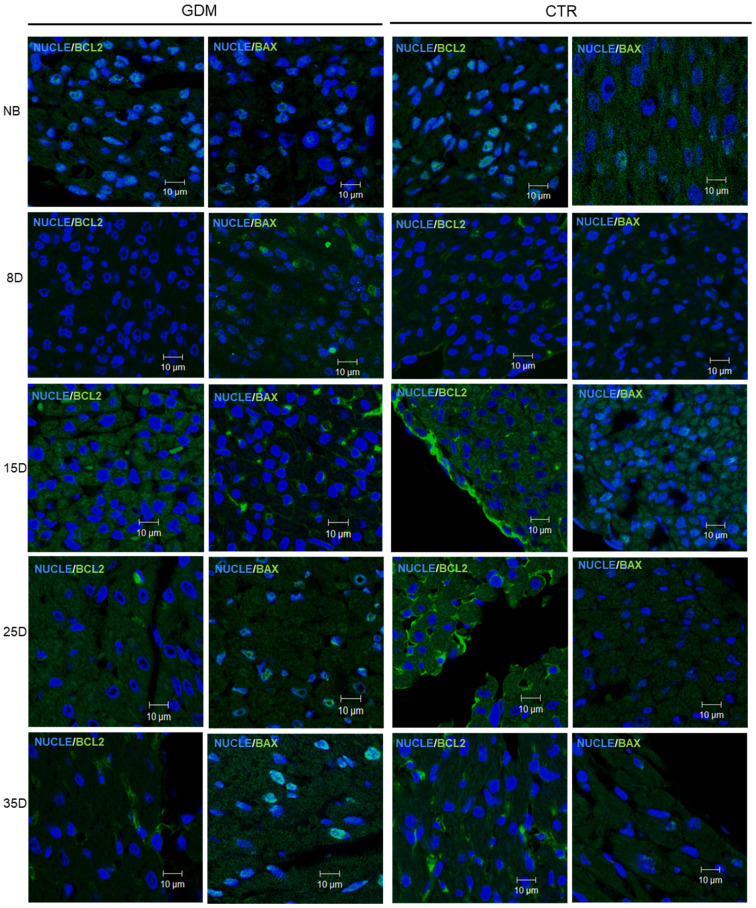
Representative 40× confocal immunofluorescence micrographs of Bax and Bcl-2 in postnatal hearts from control (CTR) and gestational diabetes (GDM) groups. Bax signal intensity increased from day 15 onward in GDM pups, whereas Bcl-2 showed a progressive reduction beginning on day 8 compared with controls. *n* = 5 litters per group.

A significant increase in the Bax/Bcl-2 ratio, an established indicator of a pro-apoptotic shift, was detected in the left ventricle of GDM offspring from 15 days of age onwards (Figure 3C and Figure 4). Interestingly, there was a modest but significant correlation between the Bax/Bcl-2 ratio and the extent of fibrosis (r = 0.303, *p* = 0.043). This suggests that the early activation of apoptotic signalling may contribute to, or occur alongside, the development of fibrotic remodelling under gestational metabolic stress.

### 2.7. GDM Affects SIRT1 and SIRT7 Expression in the Cardiac Tissue of Offspring

**Figure 5:**
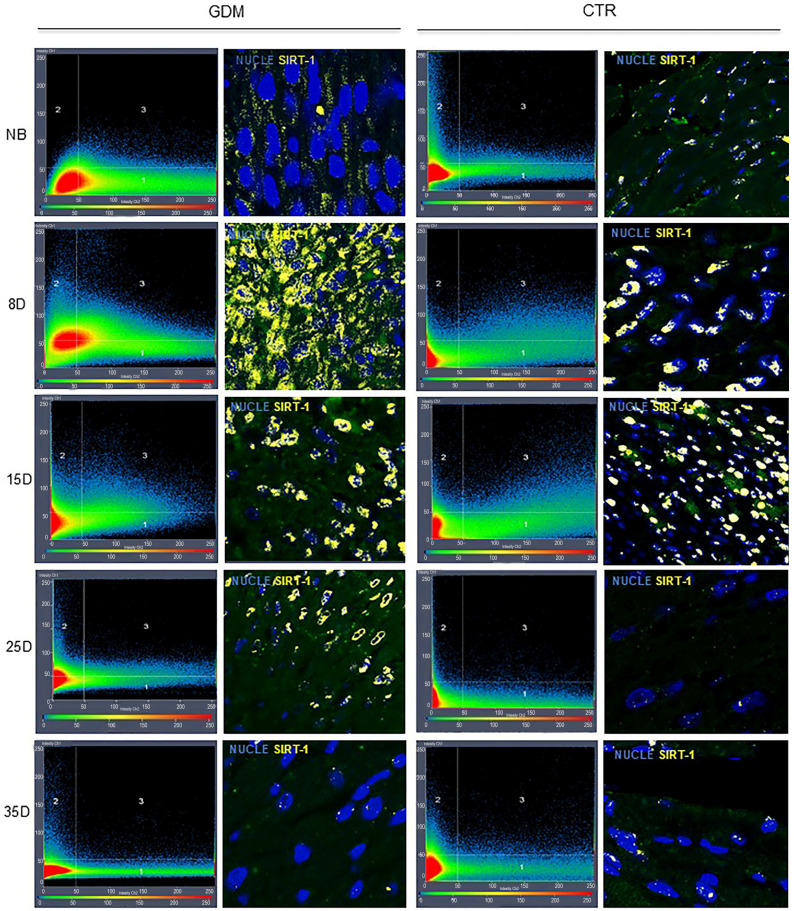
Representative 40× confocal immunofluorescence micrographs of SIRT1 in postnatal hearts from control (CTR) and gestational diabetes (GDM) groups. Histograms for each group are shown in the left columns, and nuclear SIRT1 localization is indicated in quadrant 3 (yellow). *n* = 5 litters per group; 1–2 pups per litter were analyzed at each postnatal age.

**Figure 6:**
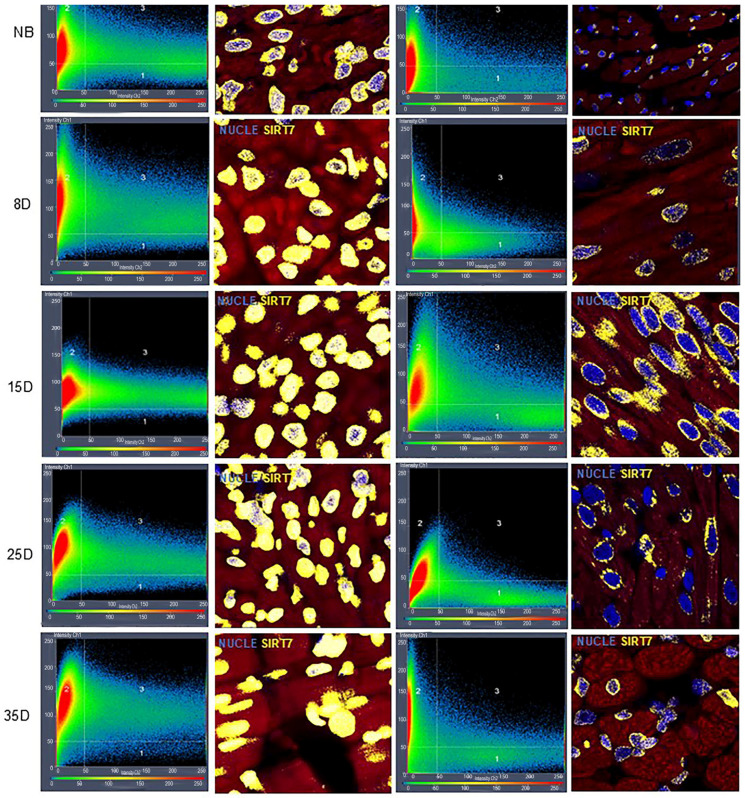
Representative 40× confocal immunofluorescence micrographs of SIRT7 in postnatal hearts from control (CTR) and gestational diabetes (GDM) groups. The SIRT7 signal (yellow) was detectable from newborn (NB) to day 35 in GDM pups. Histograms for each group are shown in the left column, and nuclear SIRT7 localization is indicated in quadrant 3. *n* = 5 litters per group; 1–2 pups per litter were analyzed at each postnatal age.

Immunofluorescence data revealed only a non-significant increase in SIRT1 localization in the hearts of 8D offspring born with gestational diabetes. No changes were observed in offspring of other ages (Figure 3D and Figure 5). Histogram results revealed that SIRT7 expression remained elevated from birth to 35 days in the GDM offspring (Figure 3E and Figure 6). Interestingly, SIRT1 expression was strongly correlated with SIRT7 expression (r = 0.613, *p* = 0.00). Additionally, both SIRT1 and SIRT7 levels were moderately correlated with fibrosis (SIRT1r = 0.405, *p* = 0.006; SIRT7r = 0.339, *p* = 0.023).

### 2.8. Epigenetic Crosstalk Between p53, miR-34a, SIRT1, and SIRT7 in Offspring Exposed to GDM

**Figure 7:**
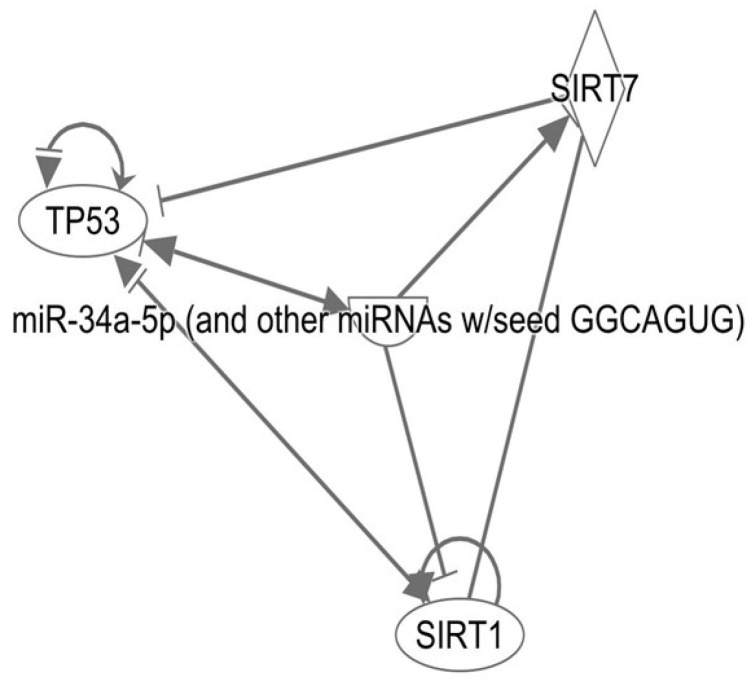
The interaction map was generated using Qiagen’s IPA program (https://digitalinsights.qiagen.com/products-overview/discovery-insights-portfolio/analysis-and-visualization/qiagen-ipa/ (accessed on 13 February 2026). The network is presented as a non-causal hypothesis based on the temporal expression patterns of *miR-34a* (day 8), *p53* (day 15) and sirtuin regulation observed in offspring exposed to gestational diabetes during pregnancy. This sequence has been integrated within the proposed *miR-34a–p53*–sirtuin regulatory axis.

Through IPA, interactions among p53, miR-34a, SIRT1, and SIRT7 were established (Figure 7). Notably, miR-34a regulates p53 expression; however, this miR blocks SIRT1 expression but activates SIRT7. Although p53 can self-regulate, in cardiac tissue from offspring of different ages exposed to GDM, both SIRT1 and SIRT7 inhibited p53 activity. These findings reveal that epigenetic dysregulation is dependent on prenatal exposure, which could contribute to increased susceptibility to apoptosis in key tissues during postnatal development.

## 3. Discussion

The results of this study support the idea that GDM acts during early development to produce long-lasting cardiac effects, consistent with DoHaD theory. Previous work has shown that intrauterine exposure to hyperglycemia, hyperinsulinemia, and oxidative stress can disrupt processes essential for heart formation, including cardiomyocyte growth, mitochondrial maturation, and epigenetic programming [3,20]. In this context, our results demonstrate that GDM has a significant impact on the structure and function of the heart during the early postnatal period, a critical window for establishing the adult cardiac phenotype. This period was characterized by altered *p53–miR-34a*–SIRT1/7 axis regulation. Dysregulation of this axis was associated with impaired cardiomyocyte growth, increased fibrosis, and elevated apoptosis—changes linked to structural and functional abnormalities in the offspring, that manifested as initial transient left ventricular hypertrophy followed by progressive remodeling with features consistent with dilated cardiomyopathy.

LVH was observed in the offspring of mothers with GDM from birth until they were 15 days old, after which the overall cardiac area was comparable to controls, and ventricular lumen dimensions had normalized. Cardiomyocyte growth remained active at this stage, but there was no evidence of fibrosis, indicating ongoing maturation without fibrotic remodeling. These findings confirm previous reports of LVH in offspring exposed to GDM [21]. The observed structural changes suggest a stiffer, more fibrotic environment during early development, possibly associated with fetal myocardial remodeling due to the adverse fetal environment. Hyperglycemia is known to promote cardiac remodeling through multiple mechanisms—oxidative stress, pro-fibrotic signaling, apoptosis, and altered cardiomyocyte metabolism—resulting in increased fibrosis, as demonstrated in cardiomyocytes from diabetic patients and in the offspring of mothers with T2D [9,21,22,23,24,25]. Concurrently, the activation of the *miR-34a*/SIRT1/*p53* axis has been associated with the regulation of cell survival and apoptotic pathways in metabolic diseases [19,26,27]. This suggests that metabolic stress and epigenetic regulators may converge to disrupt early cardiac development.

The temporal expression patterns of *miR-34a* and *p53* in the offspring of GDM mothers suggest a coordinated but stage-specific regulatory response in the developing heart. The marked increase in *miR-34a* observed on day 8, together with the slight elevations detected in newborns and 25-day-old GDM offspring, indicates that hyperglycemia-associated stress may activate *miR-34a* early in postnatal life. Furthermore, the increase in miR-34a on day 8 preceded the increment in *p53* expression on 15D. This temporal sequence suggests that *miR-34a* acts as an upstream modulator of *p53* within the fetal cardiac stress response pathway activated by gestational diabetes mellitus. In contrast, p53 expression was reduced in 8-, 25-, and 35-day-old GDM offspring, suggesting that the increment on day 15 represents a transient and highly specific activation rather than a sustained response. This transient peak may reflect a critical window during which cardiomyocytes are particularly sensitive to metabolic or oxidative stress associated with maternal diabetes.

Furthermore, exposure to a glucose-rich intrauterine environment was associated with decreased levels of SIRT1 and increased levels of SIRT7 in the offspring’s left ventricle. Together with the day 8 elevation of *miR-34a* preceding the transient *p53* surge on day 15, these findings suggest that gestational diabetes can influence multiple components of the *miR-34a-p53*-sirtuin regulatory network. As shown in the interaction map derived from the IPA, this network is presented as a hypothetical framework, not a causal one, based on the observed expression patterns. This coordinated molecular response could be responsible for the persistence of left ventricular hypertrophy up to day 15, suggesting that early postnatal cardiac remodeling is strongly influenced by metabolic stress experienced in the womb.

These findings suggest the activation of cellular stress pathways and adverse remodeling, where the loss of SIRT1—a cardioprotective sirtuin—facilitates the hyperactivation of p53 and apoptosis in neonatal cardiomyocytes. Previous studies have shown that SIRT1 deficiency worsens cardiac injury during ischemia–reperfusion [28], whereas its increase inhibits cardiomyocyte apoptosis in response to oxidative stress [29]. This coincides with the increase in the Bax/Bcl-2 ratio observed in 15-day-old pups. It is known that apoptosis contributes to dysfunction and tissue loss in various cardiomyopathies [21,30]. Furthermore, the regulation of SIRT1 by miR-34a has been associated with cardiac damage in various pathologies, including acute myocardial infarction, diabetic cardiomyopathy [31], and cardiac aging [32]. In addition, it contributes to impaired cardiac development under diabetic conditions [33]. These findings highlight the cardioprotective role of SIRT1 and reinforce its importance in conditions characterized by redox imbalance and cellular stress.

On the other hand, the increase in SIRT7 expression could represent an adaptive response to preserve cell viability under metabolic stress, although it contributes to a hypertrophic phenotype and subsequently to the development of cardiomegaly or dilated cardiomyopathy, which was previously reported in 30% of cases of children of women with GDM [7,34]. However, the left ventricular hypertrophy reported in this study was transient. These findings reveal that epigenetic dysregulation is dependent on prenatal exposure to hyperglycemia, which could increase the susceptibility of key tissues to apoptosis during postnatal development. However, the central role of SIRT7 in regulating this axis is unknown. Few studies have shown that SIRT7 is a key regulator of p53, modulating its activity according to cellular needs or context. Mice lacking both SIRT7 and p53 die prematurely, whereas p53-deficient mice develop tumors. By contrast, mice lacking SIRT7 develop cardiac hypertrophy and inflammatory cardiomyopathy, while 200% of SIRT7-deficient primary cardiomyocytes undergo apoptosis [17]. Consistently, SIRT7 deficiency in cardiomyocytes worsens hypertrophic remodeling in response to pressure overload. SIRT7’s mechanism of action involves directly interacting with and deacetylating GATA4, which limits its transcriptional activity and the expression of hypertrophy-related genes [35]. On the other hand, SIRT7 appears to play a central role in the regulation of glucose and lipid metabolism [36]. These findings suggest that SIRT7 acts as a compensatory mechanism to reverse hypertrophy, but when metabolic alterations in offspring are maintained, cardiomyopathy occurs. The data obtained suggest that newborns of mothers with gestational diabetes mellitus exhibit persistent left ventricular hypertrophy until 15 days of age. Therefore, this period could be considered critical, as it is associated with significant interstitial fibrosis, which is correlated with the expression of *miR-34a*.

However, more research is needed to understand the role of SIRT7 in the development of cardiomyopathy in offspring of mothers with gestational diabetes. One approach would be to evaluate SIRT7 function by examining the impact of pharmacological modulation and functional assays on fibrosis, apoptosis, and hypertrophic remodeling in offspring exposed to gestational diabetes. Alternatively, studies could examine the effects of inactivating or overexpressing SIRT7 in cardiomyocytes.

### Limitations

This study has several limitations. The STZ-induced gestational diabetes model only partially reproduces the clinical complexity of the human disease. Functional cardiac assessments and evaluations in adult offspring were not performed, which restricts the ability to draw conclusions about long-term cardiovascular outcomes. Furthermore, analyses were limited to the left ventricle, without systemic metabolic profiling or sex-specific comparisons. Fibrosis was assessed using hematoxylin and eosin (H&E) staining rather than collagen-specific methods, which could lead to an underestimation of extracellular matrix remodeling. Furthermore, while key regulators such as *p53*, SIRT1, SIRT7 and *miR-34a* were investigated, other pathways involved in cardiac programming were not explored. No experimental interventions were included to establish causal relationships.

## 4. Materials and Methods

Ten adult female and three adult male Sprague–Dawley rats (320 ± 20 g) from the animal facility of the National Medical Center SXXI, IMSS, were used. The animals received a Formulab diet 5008 and water ad libitum and were kept under 12 h light/dark conditions, in accordance with the recommendations of the Mexican Official Standard NOM-062-ZOO-1999 [37]. This study was approved by the Ethics Committee and Health Research of the Instituto Mexicano del Seguro Social, Mexico (R-2023-3601-124; 6 June 2024) and the Hospital Infantil de México Federico Gómez (HIM/2021/049; 23 September 2021).

### 4.1. Induction of Gestational Diabetes

A vaginal smear was obtained in the morning to identify the stage of the estrous cycle. Estrus females were housed with a male. The following morning, pregnancy was confirmed by the presence of sperm in the smear, which was recorded as day “0”. The females were randomly divided into a gestational diabetes group (GDM) and a control group (CTR). On the fifth day of gestation, 50 mg/kg of STZ in citrate buffer (pH 4.5) was administered intraperitoneally. Two days later, females with glucose levels greater than 120 mg/dL (average glucose value: 213 ± 85 mg/dL) (ACCU-CHEK Performa, Roche S.A. de C.V., Ciudad de México, Mexico) were included in the gestational diabetes mellitus group [38]. The control group received only citrate buffer. A total of 10 dams were included in the study (*n* = 5 control and *n* = 5 gestational diabetes). Each dam produced an average of 10 pups, and the entire litter was considered the experimental unit. Pups from each litter were distributed across the predefined postnatal age groups (newborn [NB], 8 days [8D], 15 days [15D], 25 days [25D], and 35 days [35D]), with 1–2 pups per litter assigned to each age. Each pup was used at a single time point only. Therefore, the sample size for each postnatal age corresponds to the number of litters per experimental group (*n* = 5), not the number of pups.

### 4.2. Sample Collection

Pups at different stages of life were anesthetized with xylazine (10 mg/kg; Xylazine Injectable Aranda, Aranda, Jalisco, Mexico) and Zoletil^®^ 100 (20 mg/kg; Vibarc S.A., Carros, France). At least four hearts per group and time point were fixed in 3.5% formalin for morphometric, histological and immunofluorescence studies. For qPCR analysis, the left ventricles (LVs) of four to five individuals were frozen at −80 °C until required.

### 4.3. Morphometric Analysis

The fixed samples were washed with PBS and dehydrated through a graded series of alcohols (30% to 70%). The atria, inflow tracts, and outflow tracts were removed from cross-sections. The ventricular portion was photographed to measure the thickness of the interventricular septum (IVS) and the free walls of both ventricles (RVL and LLV). The micrographs were analyzed using ImageJ software version 1.54K (https://imagej.nih.gov/ij/docs/faqs.html#cite (accessed on 7 January 2026)).

### 4.4. Histological Analysis of the Left Ventricle

The ventricles were dehydrated and embedded in paraffin. Five-micron sections were prepared. The slides with the sections were stained with hematoxylin/eosin and photographed under a light microscope (Medline Scientific™ CETI Steddy Stereo Trinocular Microscope, Shrewsbury, UK, 10× and 40×). The interstitial space index was quantified from micrographs using the ImageJ program (http://imagej.net).

### 4.5. Indirect Immunofluorescence

After the sample slides were deparaffinized and hydrated, antigen recovery was performed using a citrate solution, and specificity was inhibited with a universal protein blocker. The cardiomyocyte organization and area were analyzed using wheat germ agglutinin (WGA) 488.

On the other hand, the antibodies for SIRT1, SIRT7, Bax, and Bcl-2 (Santa Cruz Biotechnology, Inc., Dallas, TX, USA) were evaluated. Primary antibodies were detected using a secondary anti-mouse or anti-rabbit antibody (Santa Cruz Biotechnology, Inc.), as appropriate, coupled to fluorophore 488 or 594. In all cases, the nuclei were stained with DRAQ-7. A Zeiss LSM 510 confocal microscope (Zeiss, Oberkochen, Germany) with Zen 2009 software version 5,5,0,443, and a 1.5-pinhole lens was used to analyze all the samples. Three individuals were evaluated per group. Five micrographs were captured per slice of the left ventricular wall at 40× magnification with 1.5× zoom.

### 4.6. Quantitative Real-Time PCR

A pull was performed for RNA extraction from the left ventricle of three frozen hearts per group using the conventional TRIzol technique (TRIzol™, Invitrogen, Waltham, MA, USA). To evaluate p53 mRNA (5′-GTTCATCAGCTGGGCACCTA-3′ and 5′-CAAGTAGTCGACCCGTGGAT-3′), cDNA was synthesized from total RNA. SYBR-green universal PCR was used for amplification. Master Mix 2X (Applied Biosystems, Foster City, CA, USA) was used under the following conditions: denaturation for 1 cycle at 50 °C for 2 min, 1 cycle at 95 °C for 10 min, 40 cycles of annealing (95 °C for 15 s, 60 °C for 60 s), and extension for 1 cycle at 25 °C for 10 min. GAPDH was used as the constitutive gene (5′-ATGGTCAAGGTCGGTGTGAAC-3′ and 5′-GAAGGCAGCCCTGGTAACC-3).

On the other hand, the expression of miR-34a was evaluated (5′-UGGCAGUGUCUUAGCUGGUUGU-3′) using the TaqMan^®^ MicroRNAssays system (Applied Biosystems, Foster City, CA, USA). Amplification was performed under the following conditions: denaturation for 1 cycle at 95 °C for 10 min and 40 cycles of annealing (95 °C for 60 s, 60 °C for 60 s, and 95 °C for 30 s). RNU6B (5′-CGCAAGGATGACACGCAAATTCGTGAAGCGTTCCATATTTTT-3′) was used as the constitutive miRNA. qPCR-RT was performed on an Mx30005Tm system with MxPro software version 3.00 (Agilent Technologies, Santa Clara, CA, USA). Triplicate studies were performed in both cases, and the obtained Ct values were analyzed using Livak’s 2^−ΔΔCt^ method [39]. The relative expression of each problem group was determined by comparing it with the control group according to age.

### 4.7. Analysis of Molecules of Interest

Using Qiagen’s IPA program (https://digitalinsights.qiagen.com/products-overview/discovery-insights-portfolio/analysis-and-visualization/qiagen-ipa/ version 159584291 (accessed on 13 February 2026)), a comprehensive analysis of the set of molecules of interest was performed to identify possible regulatory interactions and build a mechanistic hypothesis about how the activation or inhibition of an upstream regulator modulates the expression of the target molecule and its repercussions on critical signaling pathways associated with cardiac damage.

### 4.8. Statistical Analysis

Immunofluorescence micrographs obtained with a confocal microscope were analyzed using LSM Zen 2009, version 5,5,0,443. Cytofluorograms and scatter plots were employed to determine the nuclear localization of the primary antibodies (SIRT1 and SIRT7). The degree of colocalization between channel 1 (nucleus) and channel 2 (SIRT-1 or SIRT-7) was used to determine the overlap coefficient in the confocal micrographs, as shown in quadrant 3. Each fluorescence has 256 intensity levels (8 bits) and is represented by a two-dimensional graph or a 256 × 256 intensity-level dot plot with a 50 × 50 background-intensity-level criterion, as indicated by the program. Statistical analysis was performed using the average value obtained in quadrant 3 per group by analyzing three independent individuals [12].

The data collected were analyzed using IBM SPSS Statistics 26.0. Quantitative data are presented as means ± standard deviations and a *p*-value < 0.05 was considered statistically significant. Student’s *t*-test for independent samples was used to compare differences between groups according to age. Additionally, Pearson’s correlation was used to establish the relationships between the variables. Age was analyzed independently using data from at least three individuals. Statistical significance was indicated when *p* < 0.05.

A one-way analysis of variance (ANOVA) was used for comparisons between age groups, employing the Shapiro–Wilk normality test and Levene’s test to determine homogeneity. Student’s *t*-test for independent samples was used for comparisons within age groups. Additionally, the Spearman rank correlation coefficient (r) was calculated to assess correlation. Subsequently, Stata version 19.0 was used to perform a mixed-models analysis of repeated measures (MMRM). Statistical significance was set at *p* < 0.05.

## Data Availability

The original contributions presented in this study are included in the article. Further inquiries can be directed to the corresponding author.

## Abbreviations

The following abbreviations are used in this manuscript:

Bax	Bcl-2-associated X protein apoptotic protein
Bcl-2	B-cell lymphoma 2 Anti-apoptotic protein
CTR	Control group
DoHaD	Developmental Origins of Health and Disease
GATA4	Four–zinc finger transcription factor
GDM	Gestational diabetes mellitus
H&E	Hematoxylin and eosin
IPA	Software
LV	Left ventricle
LVH	Left ventricular hypertrophy
LVWL	Left ventricular wall thickness
miR	microRNA
NB	Newborn
P53	P53 protein
RT-qPCR	Quantitative reverse transcription-PCR
SIRT1	Sirtuin 1
SIRT7	Sirtuin 7
